# Association between different measurements of blood pressure variability by ABP monitoring and ankle-brachial index

**DOI:** 10.1186/1471-2261-10-55

**Published:** 2010-11-05

**Authors:** Estefânia Wittke, Sandra C Fuchs, Flávio D Fuchs, Leila B Moreira, Elton Ferlin, Fábio T Cichelero, Carolina M Moreira, Jeruza Neyeloff, Marina B Moreira, Miguel Gus

**Affiliations:** 1 Division of Cardiology, Hospital de Clínicas de Porto Alegre, Brazil; 2 Postgraduate Program in Medicine: Cardiology, School of Medicine, Universidade Federal do Rio Grande do Sul, Brazil; 3 Postgraduate Program in Epidemiology, School of Medicine, Universidade Federal do Rio Grande do Sul, Brazil

## Abstract

**Background:**

Blood pressure (BP) variability has been associated with cardiovascular outcomes, but there is no consensus about the more effective method to measure it by ambulatory blood pressure monitoring (ABPM). We evaluated the association between three different methods to estimate BP variability by ABPM and the ankle brachial index (ABI).

**Methods and Results:**

In a cross-sectional study of patients with hypertension, BP variability was estimated by the time rate index (the first derivative of SBP over time), standard deviation (SD) of 24-hour SBP; and coefficient of variability of 24-hour SBP. ABI was measured with a doppler probe. The sample included 425 patients with a mean age of 57 ± 12 years, being 69.2% women, 26.1% current smokers and 22.1% diabetics. Abnormal ABI (≤ 0.90 or ≥ 1.40) was present in 58 patients. The time rate index was 0.516 ± 0.146 mmHg/min in patients with abnormal ABI versus 0.476 ± 0.124 mmHg/min in patients with normal ABI (P = 0.007). In a logistic regression model the time rate index was associated with ABI, regardless of age (OR = 6.9, 95% CI = 1.1- 42.1; P = 0.04). In a multiple linear regression model, adjusting for age, SBP and diabetes, the time rate index was strongly associated with ABI (P < 0.01). None of the other indexes of BP variability were associated with ABI in univariate and multivariate analyses.

**Conclusion:**

Time rate index is a sensible method to measure BP variability by ABPM. Its performance for risk stratification of patients with hypertension should be explored in longitudinal studies.

## Background

The biomechanics of vascular damage induced by higher blood pressure include circumferential, axial and shear stresses [1]. In addition, blood pressure variation by time (blood pressure variability) could play an additional role in the development of endothelial dysfunction and atherosclerosis [2,3]. This influence has been demonstrated in experimental models [4]. The independent association between higher blood pressure variability and clinical outcomes has been also identified in clinical [5,6] and epidemiological models [7,8]. The precise assessment of blood pressure variability is only possible with beat-to-beat BP recording [3,9], but the development of non-invasive ambulatory blood pressure monitoring (ABPM) opened the opportunity to estimate blood pressure variability through various indexes [7,8,10-13]

Despite the growing interest in using these parameters for assessment of cardiovascular prognosis, it is still unclear if they add substantial information over blood pressure values and if there is superiority of any index in this regard. Guidelines still do not recommend the inclusion of measurements of blood pressure variability on the report of ABPM examinations [14,15].

The ankle brachial index (ABI) has been considered a marker of macrovascular atherosclerotic lesion [16-23], and has been proposed to evaluate the integrity of major arteries in high risk patients [24]. Values < 0.90 and ≥ 1.4 are associated with cardiovascular risk [24,25]. As far we know, the association between abnormal ABI and blood pressure variability was not described to date, and may be useful to explore the performance of various indexes of blood pressure derived from ABPM. In this report we present the association, in hypertensive patients, between abnormalities in the ABI and three indexes of blood pressure: the standard deviation (SD) of mean BP [7,8], the coefficient of variability [6] and time rate index [10].

## Methods

This cross-sectional study was conducted in the Hypertension Clinic of the Division of Cardiology of Hospital de Clínicas de Porto Alegre (Porto Alegre, Brazil). The study was approved by the Ethics Committee of our Institution, which is accredited by the Office of Human Research Protections as an Institutional Review Board, and all patients signed a written informed consent for participation.

The study population was selected from a consecutive sample of patients screened for participation in randomized clinical trial of approaches to improve adherence in patients with hypertension (MONITOR study/NCT00921791). Patients were included in this analysis if they had: [1] history of hypertension and were aged between 18 to 80 years; [2] absence of history or clinical evidence of severe complications related to hypertension (coronary artery disease, heart failure, cerebrovascular disease, end stage renal failure); [3] absence of clinical suspicion or laboratory evidence of secondary hypertension; [4] agreed to participate in the study and had the ability to provide the free and informed consent; [5] possibility to perform ABI measurement (without amputation or ulcers of the lower limb).

Patients underwent to an extensive demographic and clinical baseline data collection, including the assessment of education, alcohol and tobacco consumption. Patients with a history of angioplasty or coronary revascularization, carotid endarterectomy, myocardial infarction, angina, heart failure, stroke, and transient ischemic attack were considered as having cardiovascular disease. The prevalence of peripheral arterial occlusive disease (PAD) was defined as presence of intermittent claudication or angioplasty of lower limb vessels. Diabetes mellitus was defined as fasting plasma glucose ≥ 126 mg/dl or use of antidiabetic drugs. BP was measured with an office aneroid sphygmomanometer and the mean values were estimated after an average of 6 measures in 3 different visits according to guidelines [26,27]. Patients with BP within normal values but taking BP drugs and those with severe hypertension (≥ 180/110 mmHg) or evidence of clinical disease were classified on the occasion of the first visit. Hypertension was defined as a mean blood pressure ≥ 140/90 mm Hg or use of antihypertensive medications. During the first visit ABI was measured according to standard protocol [24] by trained physicians using a device for Doppler ultrasound (Doppler vascular laptop, model DV 610, with 10 MHz frequency). A mercury manometer and appropriate cuffs for measurement of brachial blood pressure and lower limbs were used.

The ABI was defined as the ratio between the highest systolic blood pressure of the ankle (posterior tibial or dorsalis pedis arteries) and higher systolic pressure of the arm (right or left) [24]. ABI was calculated for each leg as the ratio between the average of two measures of pressure on each limb. The cutoff point for diagnosis of peripheral arterial disease (PAD) was ABI ≤ 0.90 or ≥ 1.40 [24,25].

All individuals were submitted to ABPM in a normal working day (Spacelabs 90207, Spacelabs, Redmond, WA). Readings were obtained at intervals of 15 minutes during the day and 20 minutes on the night of 24 hours throughout the period studied. Patients with less than 6 and 18 measures during the night and the day periods respectively were excluded from further analysis [14]. Based on the results of ABPM, the mean 24-hour systolic (SBP) and diastolic (DBP) blood pressures were calculated for each patient. We calculated three different parameters of systolic pressure variability: the standard deviation of mean (SD), coefficient of variability (CV = SD/mean pressure X100%) and rate of change in SBP over time (mmHg/min) defined as the first derivative values of SBP by time (time rate index). This index allows the calculation of the sum of angular coefficients and aims to measure how fast or how slow and which direction SBP values change. The measure was calculated using the following formula [10]:

$$R=|\bar{r}|=\frac{\sum_{i=1}^{N-1}\left|ri\right|}{N-1}$$

Where r is the rate of BP variability over time (considering the differences between blood pressure measurements in each time intervals) and N is the number of recordings.

### Statistical Analyses

The groups for comparison were defined by the presence or absence of an abnormal ABI: values ≤ 0.9 or ≥ 1.4 in any lower limb. Comparisons were tested by Pearson's chi-square test, unpaired Student t test, and Mann-Whitney test. Logistic regression models and multiple linear regression were used to evaluate the association between parameters of variability of 24-hour SBP and ABI. Age, 24-hour SBP and diabetes were included in models. Three patients with ABI ≥ 1.4 in any leg were not included in the multiple linear models. All values are presented as mean and standard deviation, with their confidence intervals of 95% (CI). Values of P <0.05 were considered significant. The areas under (AUC) the Receiver-operating characteristic curve (ROC) of different 24-hour SPB variability indices were calculated to compare the performance of these parameters in the prediction of abnormal ABI [28].

Data were analyzed using the program Statistical Package for Social Science (SPSS version 14.0, Il, USA).

## Results

A total of 482 patients were evaluated, being 57 excluded because of lack of ABPM or ABI data, leading to the analysis of 425 patients (88% of those screened). The characteristics of the sample are presented in Table 1. Abnormal ankle-brachial index (ABI ≤ 0.90 and/or ≥ 1.4) was detected in 58 patients (13.6%). Participants with abnormal ABI were older, had higher 24-hour SBP and a higher proportion of diabetics (Table 1). Among the indexes of blood pressure variability, only the time rate index was statistically different between the groups (Table 1**)**. Standard deviation and coefficient of variability calculated separately for the daytime and nighttime periods were not associated with abnormal ABI also.

**Table 1:** Distribution of selected characteristics in the whole sample and by the presence of an abnormal ABI

	Total sample (n = 425)	Abnormal ABI* (n = 58)	Normal ABI (n = 367)	P**
Age (years)	57.4 ± 12.1	63.6 ± 10.6	56.4 ± 12.0	<0.001
Male	131 (30.8%)	21 (36.2%)	110 (30%)	0.34
Body mass index (kg/m^2^)	30.8 ± 5.8	29.7 ± 5.5	30.9 ± 5.9	0.12
White	280 (65.9%)	34 (58.6%)	246 (67%)	0.21
Alcohol abuse (> 30 g/day)	35 (8.2%)	6 (10.3%)	29 (7.9%)	0.53
Current Smoking	111 (26.1%)	16 (27.6%)	95 (25.9%)	0.44
(Years at school)				0.005
≤ 4 years	124 (29.2%)	26 (44.8%)	98 (26.7%)	
> 4 years	301 (70.8%)	32 (55.2%)	269 (73.3%)	
Diabetes mellitus	94 (22.1%)	20 (34.5%)	74 (20.2%)	0.01
Duration of hypertension (years)				0.01
≤ 10 years	212 (49,9%)	191 (53.5%)	21 (36.2%)	
> 10 years	203 (47.8%)	166 (46.5%)	37 (63.8%)	
Cardiovascular disease	119 (28%)	19 (32.8%)	100 (27.2%)	0.38
Office SBP (mmHg)	152.4 ± 25.9	156.9 ± 28.8	151.7 ± 25.5	0.16
Office DBP (mmHg)	88.7 ± 14.8	85.6 ± 14.7	89.2 ± 14.8	0.08
24-hour SBP (mmHg)	136.8 ± 17.3	141.0 ± 18.0	136.2 ± 17.1	0.04
24-hour DBP (mmHg)	80.9 ± 12.5.	78.8 ± 12.9	81.3 ± 12.5	0.13
Use of antihypertensive	316 (74.4%)	45 (77.6%)	271 (73.8%)	0.54
N° antihypertensive drugs (P25%-75%)	1 (1-1)	2 (1-3)	2 (0-3)	0.19
Time Rate SBP index (mmHg/min)	0.476 ± 0.124	0.516 ± 0.146	0.469 ± 0.119	0.007
SD SBP (mmHg)	12.7 ± 3.8	13.2 ± 4.7	12.6 ± 3.7	0.26
CV SBP (%)	9.3 ± 2.7	9.3 ± 2.6	9.3 ± 2.9	0.91

The independent association between the time rate index and abnormal ABI was assessed by logistic regression models and multiple linear regression (considering the right and left legs). The association was independent of age (Table 2-model 1), but was lessened when 24-hour SBP and diabetes were added to the model (Table 2- model 2). With the exclusion of three individuals with abnormal ABI because of higher values, the risk ratios increased and were almost significant in the full model: RR 18.7 (95% CI: 1.96-198.5; P = 0.01) when adjusted for age and RR 10.7 (95% CI: 0.89-129.1; P = 0.06) when adjusted for age, diabetes and 24-hour systolic blood pressure. Models with the quadratic and cubic term of the time-rate did not improve the intensity of association, therefore excluding a relevant non-linear association. The SD of 24-hour SBP and the CV were not associated with abnormal ABI either in the model 1 (P = 0.46 for SD of 24-hour SBP and P = 0.90 for CV, respectively) or in the second model (P = 0.70 for SD and P = 0.92 for CV, respectively). A multiple linear regression model of the minimum ABI showed no association with any of the variables examined, possibly due to a restricted range of response. However, the time-rate index was associated with the ABI separately in the right and left legs, and with the mean ABI for the patient (table 3), adjusting for age, diabetes and 24-hour SBP. The introduction of 24-ABPM pulse pressure in the models instead of systolic blood pressure did not change the independent association between the time rate index and ABI. SD of 24-hour SBP and the coefficient of variability were not associated with ABI in this model (P higher than 0.05 for both indexes and both legs).

**Table 2:** Association of time rate index with abnormal ABI in logistic regression models

Model 1	RR (95% CI)	P
Time Rate (mmHg/min)	6.88 (1.12-42.15)	0.04
Age (years)	1.05 (1.02-1.07)	<0.001

**Model 2**	**RR (95% CI)**	**P**

Time Rate (mmHg/min)	5.84 (0.52-65.0)	0.15
Age (years)	1.05 (1.03-1.08)	<0.001
24-hour SBP (mmHg)	1.01 (0.99-1.03)	0.41
Diabetes mellitus	1.71 (0.92-3.18)	0.09

**Table 3:** Association between the time-rate index and ABI in multiple linear regression model

	Beta	**S.E**.	P
**Right lower limb**			

Time Rate SBP (mmHg/min)	-0.15	0.06	0.003
24-hour SBP (mmHg)	-0.09	< 0.001	0.09
Age (years)	-0.11	0.001	0.02
Diabetes Mellitus	-0.02	0.15	0.62

**Left leg limb**			

Time Rate SBP (mmHg/min)	-0.13	0.05	0.01
24-hour SBP (mmHg)	-0.05	< 0.001	0.28
Age (years)	-0.12	< 0.001	0.01
Diabetes mellitus	-0.01	0.01	0.82

**Mean of both legs**			

Time Rate SBP (mmHg/min)	-0.14	0.05	0.005
24-hour SBP (mmHg)	-0.08	< 0.001	0.11
Age (years)	-0.12	< 0.001	0.009
Diabetes mellitus	-0.004	0.01	0.77

Table 4 shows that only the time-rate index had statistically significant AUC values to predict abnormal ABI. The best cut-off point of the time-rate index to predict abnormal ABI was 0.478 mmHg/min. Adding the time-rate index to a model with systolic and diastolic blood pressure the C-index increased from 0.637 (95% CI 0.559 to 0.714) to 0.648 (95% CI 0.575 to 0.722).

**Table 4:** Area under the curve (AUC) of the different measurements of variability to predict an abnormal ABI

	AUC	CI 95%	P
CV SBP	0.52	0.43 - 0.60	0.70
SD SBP	0.53	0,44 - 0,61	0.48
Time Rate SBP	0.60	0.51 - 0.68	0.02

## Discussion

In this large sample of patients with hypertension screened for a randomized clinical trial we identified that nearly 14% of participants had an abnormal ABI. This prevalence is similar to that reported in population-based studies [16,17,19-21] but lower than the described in a secondary analysis of a randomized clinical trial [18]. Differences in the sample characteristics explain the variable prevalence of abnormalities in the ABI that has been reported in different studies. In the present study all participants had hypertension and almost a quarter had diabetes.

Individuals with abnormal ABI had higher blood pressure variability only if measured by the time rate index. This association was robust and independent of age when ABI was stratified in normal and abnormal. Systolic blood pressure and diabetes lowered the intensity of this association. On the other hand, when ABI was included as a dependent variable in a linear model, there was a strong and independent association with BP variability exclusively measured by the time rate index. In this regard, our findings are in agreement with those reported by Zakopoulos and colleagues, who demonstrated, in a cross-sectional study with 539 individuals, that only the time rate index was independently associated with the thickness of the carotid artery measured by ultrasound [10]. Our findings confirm that this association is independent of blood pressure levels. We had previously shown, in a secondary analysis of a randomized clinical trial [29], that the time-rate index was independent even from blood pressures changes induced by drug treatment [30].

Abnormal ABI has been identified as a marker of vascular damage by atherosclerosis [24]. Values ≤ 0.90 at rest are recognized as the cutoff for diagnosis of PAD [24] but values ≥ 1.4 are also associated with atherosclerotic disease and PAD [24,25]. Beside, it has been recognized as an independent marker of cardiovascular risk [16-23]. Various longitudinal studies have demonstrated that the severity of PAD in the lower limbs correlates with the risk of acute myocardial infarction, stroke and death from vascular causes [17-23]. Thus, the ABI is a good marker of atherosclerosis and due to its easy implementation it is increasingly being used in the daily practice for risk stratification of hypertensive patients.

The cross-sectional nature of our investigation precludes establishing temporal causality. Abnormal BP pressure variability could be responsible for the development of vascular abnormalities as well the vascular rigidity could lead to higher blood pressure variability. Longitudinal studies should be launched to identify if abnormalities in BP pressure variability precede the vascular damage detected by ABI. In face of the risks identified in cohort studies, with other indexes of blood pressure variability, it is expected that abnormalities in the ABI are favored by higher blood pressure variability. These findings can be explained by the association between the process of atherosclerosis and the hemodynamic changes that lead to vascular endothelial injury. In an elegant experiment, Cheng and colleagues demonstrated that the phenotype of endothelial injury is directly linked to the changes in blood flow [31]. Atherosclerotic plaques with a vulnerable aspect were present mainly in areas of low shear stress, while in areas of turbulent flow (or vortices) the plaques seemed stable. Interpreting these findings, Richter and Edelman stated that rather than the absolute shear stress value (higher or lower), it is the variations in blood flow that could modify the endothelial biology [32]. Therefore, parameters of pressure variation could be related to the risk of damage to a target organ in hypertension. Such findings have been demonstrated in studies using invasive measurement of blood pressure [2-5], but the challenge to incorporate the measurement of variability in daily clinical practice still remains.

Despite that the precise assessment of blood pressure variability is only possible with beat-to-beat BP recording [33], our findings expand the possibility to measure blood pressure variability by ABPM, confirming the findings of Zakopoulos et. al [10]. Different parameters of BP variability derived from ABPM have been described [11-13], but the time-rate index embodies the concept that in the analysis of blood pressure variability it is important to measure not only blood pressure differences between each measure but how fast or how slow it occurs and to which direction the blood pressure changes. Our results indicate that the time rate index may be more powerful to estimate the true variability than standard deviation from the mean and coefficient of variability. It was the only index associated with continuous and abnormal values of ABI. It was, also, the only index to have statistically significant AUC to explain about 60% of the occurrence of an abnormal ABI in this sample. It was previously demonstrated that blood pressure variability evaluated by the standard deviation and coefficient of variability using blood pressure of the nighttime period has a more intense association with cardiovascular outcomes [8,34]. This association was not evident in our study, but this may be potentially ascribed to statistical power. In a recent report from a large population cohort, BP variability assessed by standard deviation did not contribute for risk stratification beyond 24-hour BP [35]. The time-rate index may perform better, and should be also investigated in large cohort studies, including its capacity to increase the precision of prognostic estimation.

## Conclusions

In conclusion, variability of systolic blood pressure over time derived from ABPM- the time rate index is associated with the ankle-brachial index. Blood pressure variability measured by the standard deviation of 24-hour SBP and by the coefficient of variability are not associated with the ankle-brachial index. The performance of the time rate index to identify cardiovascular risk over blood pressure values should be explored in longitudinal studies.

## Competing interests

The authors declare that they have no competing interests.

## Authors' contributions

EW: participated in the design of the study, elaboration of the protocol, data collection and draft preparation. SCF: planned the investigation, supervised data collection and database preparation, run the statistical analyses and helped to prepare the draft. FDF: participated in the design of the study and data analyses and interpretation, wrote the drafts and final version. LBM: participated in the design of the study and data analyses and helped to prepare the draft. EF: developed the software for time-rate measurement and helped in the statistical analyses. FTC, CMM, JN, MBM: were responsible for data collection, including all ABI measurements and patients' interviews and examinations. MG: conceived the hypothesis, proposed the methods of investigation, supervised data collection and participated in data analyses and draft preparation. All authors read and approved the final manuscript.

## Pre-publication history

The pre-publication history for this paper can be accessed here:

http://www.biomedcentral.com/1471-2261/10/55/prepub
